# Early Nephrology Referral Reduces the Economic Costs among Patients Who Start Renal Replacement Therapy: A Prospective Cohort Study in Korea

**DOI:** 10.1371/journal.pone.0099460

**Published:** 2014-06-13

**Authors:** Jeonghwan Lee, Jung Pyo Lee, Ji In Park, Jin Ho Hwang, Hye Min Jang, Ji-Young Choi, Yong-Lim Kim, Chul Woo Yang, Shin-Wook Kang, Nam-Ho Kim, Yon Su Kim, Chun Soo Lim, CRC for ESRD investigators

**Affiliations:** 1 Department of Internal Medicine, Hallym University Hangang Sacred Heart Hospital, Seoul, Korea; 2 Clinical Research Center for End Stage Renal Disease (CRC for ESRD), Daegu, Korea; 3 Department of Internal Medicine, Seoul National University Boramae Medical Center, Seoul, Korea; 4 Department of Internal Medicine, Seoul National University College of Medicine, Seoul, Korea; 5 Department of Internal Medicine, Chung-Ang University Hospital, Seoul, Korea; 6 Department of Statistics, Kyungpook National University, Daegu, Korea; 7 Department of Internal Medicine, Kyungpook National University School of Medicine, Daegu, Korea; 8 Department of Internal Medicine, The Catholic University of Korea College of Medicine, Seoul, Korea; 9 Department of Internal Medicine, Yonsei University College of Medicine, Seoul, Korea; 10 Department of Internal Medicine, Chonnam National University Medical School, Gwangju, Korea; University of Florida, United States of America

## Abstract

**Background:**

The nature of cost-saving effects of early referral to a nephrologist in patients with chronic kidney disease (CKD) is not fully evaluated. We evaluated the health care costs before and after dialysis according to the referral time.

**Methods:**

A total of 879 patients who were newly diagnosed as having end-stage renal disease from August 2008 to June 2011 were prospectively enrolled. The early referral (ER) group was defined as patients who were referred to a nephrologist more than a year before dialysis and had visited a nephrology clinic 2 or more times. Patients whose referral time was less than a year were considered the late referral (LR) group. Information about medical costs was acquired from the claim data of the Korea Health Insurance Review and Assessment Service.

**Results:**

The total medical costs during the first 12 months after the initiation of dialysis were not different between the 526 ER patients and the 353 LR patients. However, the costs of the ER patients during the first month were significantly lower than those of the LR patients (ER vs. LR: 3029±2219 vs. 3438±2821 US dollars [USD], P = 0.025). The total 12-month health care costs before the initiation of dialysis were significantly lower in the ER group (ER vs. LR: 6206±5873 vs. 8610±7820 USD, P<0.001). In the multivariate analysis, ER significantly lowered the health care costs during the 12 months before (2534.0±436.2 USD, P<0.001) and the first month (428.5±172.3 USD, P = 0.013) after the initiation of dialysis.

**Conclusions:**

The ER of patients with CKD to a nephrologist is associated with decreased medical costs during the pretreatment period of renal replacement therapy and the early period of dialysis initiation.

## Introduction

Chronic kidney disease (CKD) is a major public health problem that is continually growing worldwide. The overall prevalence of CKD around the world is 10–16% [1]–[3]. In the United States, the prevalence estimate for CKD rose from 12.3% to 14.0% over the past 20 years [4]. Kidney function progressively declines in a proportion of CKD patients without adequate therapy, eventually progressing to devastating end-stage renal disease (ESRD) [5]. In accordance with the increasing prevalence of CKD, the prevalence of ESRD has also increased. The total treated ESRD population in the United States rose from 450,000 in 2004 to 593,086 in 2010 [4]. In Korea, the overall number of ESRD patients was 56,396, and the prevalence was 738.3 per million population, at the end of 2009 [6]. The prevalence increased by approximately 12% during the period 2000–2009.

Along with the increase in ESRD prevalence, the economic costs resulting from the care of these patients have increased. In the United States, the annual medical cost of dialysis patients borne by the Centers for Medicare and Medicaid in 2008 was 26.8 billion USD, which was 5.9% of the total costs of the entire population, and the annual increase rate reached 13.2% [7]. Among patients starting dialysis, the proportion of elderly patients and those with diabetes have increased compared with the past [8], and thus the economic burden of patients on dialysis is serious. Therefore, proper management or intervention of CKD patients is required to improve clinical outcomes and reduce medical costs.

The late referral (LR) of patients to a nephrologist in the course of CKD progression has increased the morbidity and mortality [9]–[19]. The timely referral of CKD patients to a nephrologist is associated with a higher quality of care before the start of renal replacement therapy [20]–[26] and with improved outcomes after the initiation of dialysis [27]–[31]. The increased morbidity of patients referred late to a nephrologist is likely to result in a greater usage of health care resources. Our previous investigation revealed that timely referral (1 year before dialysis) to a nephrologist integrated with education about dialysis was associated with reduced usage of temporary vascular catheters and improved survival [32]. Although early referral (ER) has been suggested to produce cost savings in addition to the health benefits for the patient, there are few reports that have directly analyzed the effects of referral time on medical costs before and after the start of dialysis. To evaluate the economic benefits of ER to a nephrologist, we investigated the difference in the economic burden according to the referral time using national health insurance claim data in a prospective cohort ESRD patients.

## Methods

### Cohort Description

This study was nested within an ongoing cohort study (Clinical Research Center for End Stage Renal Disease, CRC for ESRD) of patients with ESRD in South Korea. The CRC for ESRD is a nationwide multi-center web-based comprehensive prospective cohort of CKD patients on dialysis whose aim is to analyze the treatment effects on survival or quality of life and cost-effectiveness by dialysis modality [32], [33]. All enrolled patients are adults aged 20 years old or older and who started dialysis for ESRD without a schedule for kidney transplantation within 3 months. The patients began to be registered in July 2008, and 31 hospitals in Korea are currently participating in the CRC for ESRD cohort study. Until September 2012, a total of 1620 CKD patients who had newly started dialysis and 2917 patients who had already been on dialysis had been enrolled. All patients provided their written consent to participate voluntarily in this study. The study was approved by the institutional review board at each center [The Catholic University of Korea, Bucheon St. Mary's Hospital; The Catholic University of Korea, Incheon St. Mary's Hospital; The Catholic University of Korea, Seoul St. Mary's Hospital; The Catholic University of Korea, St. Mary's Hospital; The Catholic University of Korea, St. Vincent's Hospital; The Catholic University of Korea, Uijeongbu St. Mary's Hospital; Cheju Halla General Hospital; Chonbuk National University Hospital; Chonnam National University Hospital; Chung-Ang University Medical Center; Chungbuk National University Hospital; Chungnam National University Hospital; Dong-A University Medical Center; Ehwa Womens University Medical Center; Fatima Hospital, Daegu; Gachon University Gil Medical Center; Inje University Pusan Paik Hospital; Kyungpook National University Hospital; Kwandong University College of Medicine, Myongji Hospital; National Health Insurance Corporation Ilsan Hospital; National Medical Center; Pusan National University Hospital; Samsung Medical Center, Seoul; Seoul National University, Boramae Medical Center; Seoul National University Hospital; Seoul National University, Bundang Hospital; Yeungnam University Medical Center; Yonsei University, Severance Hospital; Yonsei University, Gangnam Severance Hospital; Ulsan University Hospital; Wonju Christian Hospital (in alphabetical order)]. All clinical investigators followed the Ethics for Medical Research and conducted this study in accordance with the guidelines of the 2008 Declaration of Helsinki.

### Study Participants

We selected and studied 1011 adult patients who were newly diagnosed as having ESRD and started dialysis between August 2008 and June 2011 from the new patient registry in the CRC for ESRD cohort. Of the 132 patients who were excluded, the data regarding visits to a nephrologist were missing for 61 patients, and data regarding the type of initial dialysis were unknown for 33 patients. In addition, 38 patients were excluded due to the lack of cost data because of an unmatched identifier. The early referral group (ER) was defined as patients who were referred to a nephrologist more than 1 year before dialysis initiation and who had visited a nephrology clinic 2 or more times; the remaining patients whose referral time was less than a year were considered the late referral group (LR) [32].

### Clinical Data Acquisition

Information about the participants was primarily collected from the data server of the CRC for ESRD for analysis. All traceable identifiers were removed prior to the analysis to ensure patient confidentiality. The clinical and laboratory data had been stored in the form of web-based medical questionnaires. The questionnaire items were filled in by data coordinators, who were trained in each center to collect patient data by a combination of chart reviews and direct interviews using a standardized form. The questionnaires included data on demographics, previous medical history, laboratory results, dialysis modality and prescription, type of permanent access used for the first dialysis, and medications. The estimated glomerular filtration rate (eGFR) was calculated by CKD-EPI equations [34]. The modified Charlson co-morbidity index (CCI) and Davies co-morbidity index (DCI) at the initiation of dialysis were recorded for each patient. The modified CCI and DCI values were calculated using the method described in a previous study [35]–[37]. The patients were asked to respond to the questionnaire when they first visited a nephrologist before starting dialysis.

### Health Care Costs

Information about the medical costs during the 1 year periods just before and after the initiation of dialysis was acquired from the claim data of the Korea Health Insurance Review and Assessment Service (HIRA). In Korea, government-initiated public health insurance is mandatory, and private health insurance without national health insurance is not allowed for direct medical costs. All populations are obligatorily registered in the national health insurance service, and almost all legitimate medical costs are controlled and reimbursed via the Korea HIRA. We calculated the medical costs by an official request for the claim data of medical costs from the Korea HIRA and data integration by the coded identifiers of patients in the CRC for ESRD. To determine the economic cost of an illness, three types of costs should be considered: direct costs, indirect costs (productivity loss), and intangible costs. In this study, we evaluated direct medical expenditures, including physician visit costs, admission costs, surgery costs, physical therapy costs, and total medicine costs. The following costs were excluded: traffic costs, which are considered a direct non-medical cost; traditional Chinese herbal medicine costs; indirect costs; and intangible costs, which cannot be directly collected through the claim data of the Korea HIRA. All of the costs were calculated in the currency unit of the Korean Won (KRW) (US$1  =  1,100 KRW in 2013).

### Statistical Analysis

In the descriptive analysis of the demographic and clinical characteristics and laboratory results, continuous variables were expressed as the mean and standard deviation, and categorical variables were described numerically with a percentage. Comparisons between the groups based on referral time were performed using a t-test for continuous variables and Chi-square or Fisher's exact tests for categorical variables when appropriate. We presented the mean with the standard error of the mean for the entire cost. The associations between medical costs and independent parameters were assessed using univariate and multivariate linear regression methods. Variables in the univariate models were selected *a priori* and by clinical significance, and included the type of referral, age, sex, type of dialysis modality, body mass index, causes of underlying kidney disease, hemoglobin level, and serum albumin level. Multivariate model included variables that were proved to be associated (P value <0.10) with the total medical costs. SAS/STAT 9.2 (SAS Institute Inc., Cary, NC, USA) was used in the analysis of the medical costs, and IBM SPSS ver. 20.0 was used to compare the demographic and clinical parameters. A P value <0.05 was considered statistically significant.

## Results

### Patients Characteristics by Referral Pattern

The patients' clinical and laboratory characteristics are summarized in Table 1. The mean age was 55.8±14.1 years old, and 59.5% of patients were male. Of the 879 patients enrolled in the cost analysis, 526 patients were in the ER group, and 353 were in the LR group. The time from referral to dialysis was significantly longer in the ER group than in the LR group (63.9±62.1 months vs. 2.7±3.4 months, P<0.001). In the LR group, the proportion of patients who visited a nephrologist less than 2 times before dialysis initiation reached up to 34.8%. The most common cause of renal disease was diabetic nephropathy in both groups.

**Table 1:** Patient characteristics by referral pattern at the time of referral and dialysis initiation.

		Total ESRD				
		Total	Early referral	Late referral	P Value	
		(*N* = 879)	(*N* = 526)	(*N* = 353)		
Age at the time of referral (year) Findings at the time of referral to nephrologist		52.5±15.1	50.6±15.1	55.4±14.6	<0.001	
Underlying kidney disease (%)					0.003	
Diabetes mellitus		48.7	49.6	47.4		
Hypertension		17.4	15.4	20.5		
Glomerulonephritis		16.4	18.7	12.9		
Polycystic kidney disease		2.5	3.5	1.1		
Others		10.5	9.3	12.1		
Unknown		4.5	3.5	5.9		
Systolic BP (mmHg)		146.3±26.8	142.9±25.1	150.2±28.1	0.001	
Diastolic BP (mmHg)		84.4±16.6	83.4±16.0	85.4±17.1	0.132	
Serum creatinine (mg/dL)		4.94±4.50	2.82±2.70	7.41±4.90	<0.001	
eGFR (mL/min/1.73 m^2^)		23.9±23.3	34.3±25.2	11.8±12.7	<0.001	
Hemoglobin (g/dL)		10.1±5.3	11.3±6.8	8.8±1.8	<0.001	
Number of visits to a nephrologist from referral to dialysis (%)					<0.001	
None		7.1	0	17.7		
1 time		6.9	0	17.1		
2 times or more		86.0	100	65.2		
Time from referral to dialysis (month)		39.2±56.6	63.9±62.1	2.7±3.4	<0.001	
Age at the time of dialysis (year)		55.8±14.1	55.9±13.8	55.6±14.6	0.761	
Gender (male, %)		59.5	59.2	60.1	0.774	
Findings at the time of dialysis						
Modified Charlson co-morbidity index		5.1±2.6	5.2±2.6	5.0±2.5	0.337	
Davies co-morbidity index2		1.0±0.9	1.0±0.9	1.0±1.0	0.836	
Systolic BP (mmHg)		139.7±22.2	138.9±22.2	140.8±22.1	0.222	
Diastolic BP (mmHg)		78.3±14.0	77.5±13.4	79.5±14.8	0.041	
BMI (kg/m^2^)		23.1±3.5	23.1±3.3	23.1±3.7	0.882	
Serum creatinine (mg/dL)		8.48±4.41	8.48±4.17	8.48±4.73	0.996	
eGFR (mL/min/1.73 m^2^)		7.6±7.4	7.3±6.6	7.9±8.4	0.214	
Hemoglobin (g/dL)		8.8±1.7	8.9±1.7	8.8±1.6	0.287	
Uric acid (mg/dL)		8.1±2.6	8.0±2.6	8.1±2.5	0.593	
Calcium (mg/dL)		7.8±1.0	7.8±1.0	7.7±1.1	0.368	
Phosphate (mg/dL)		5.4±1.8	5.4±1.8	5.5±1.9	0.672	
Total cholesterol (mg/dL)		161.3±53.6	158.6±49.9	165.5±58.6	0.063	
LDL cholesterol (mg/dL)		93.5±48.1	89.9±39.0	98.7±58.6	0.018	

Values for continuous variables are expressed as the means ± standard deviation; values for categorical variables are expressed as proportions.

BMI, body mass index; BP, blood pressure; eGFR, estimated glomerular filtration rate; HDL, high-density lipoprotein; LDL, low-density lipoprotein

At the time of the referral to a nephrologist, ages were younger, the blood pressure (BP) and serum creatinine level were lower, and the hemoglobin level and eGFR were higher in the ER group than in the LR group. At the time of dialysis initiation, most findings, including the age, sex, co-morbidity index, serum creatinine, eGFR, hemoglobin levels, calcium and phosphate levels, urate levels, and total cholesterol levels were similar in both groups. However, diastolic BP (77.5±13.4 vs. 79.5±14.8 mmHg, P = 0.041) and LDL cholesterol levels (89.9±39.0 vs. 98.7±58.6 mmHg, P = 0.018) were lower in the ER group.

### Medical Costs

**Figure 1:**
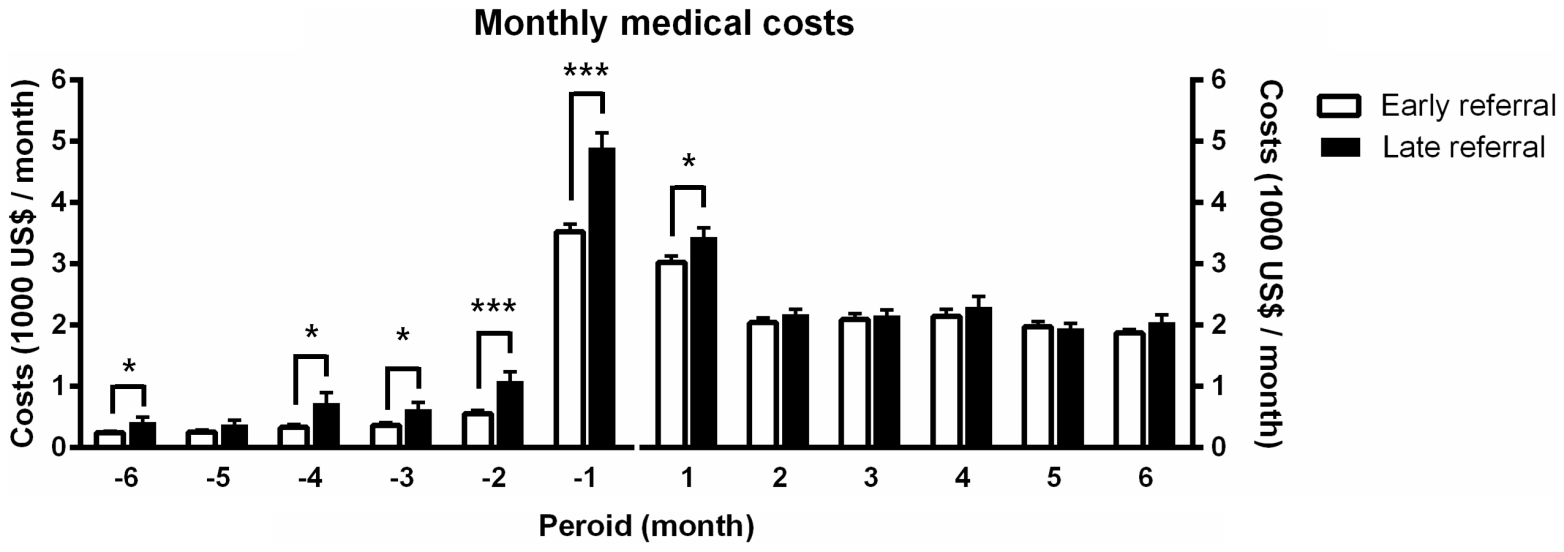
Monthly medical costs before and after the initiation of dialysis. The monthly medical costs are represented, comparing ‘early referral’ and ‘late referral’ according to the time period. The values (unit: US dollars) are expressed as the mean ± SEM. ^*^P<0.05, ^***^P<0.001.

The monthly medical costs before and after the initiation of dialysis are summarized in table 2 and figure 1. The total medical costs during the first 12 months after the initiation of dialysis were not different between the two groups (ER vs. LR: 18947±11864 vs. 19104±11501 USD, P = 0.845). However, the costs of the ER patients during the first month were significantly lower than those of the LR patients (ER vs. LR: 3029±2219 vs. 3438±2821 USD, P = 0.025). The total 12-month health care costs before the initiation of dialysis were significantly lower in the ER group (ER vs. LR: 6206±5873 vs. 8610±7820 USD, P<0.001).

**Table 2:** Medical costs during the first year before and after dialysis therapy.

	Periods	Costs (USD)		
		Early referral	Late referral	P Value
After dialysis initiation (month)	0∼1	3029±2219	3438±2821	0.025
	1∼2	2044±1679	2181±1515	0.223
	2∼3	2091±2087	2162±1669	0.597
	3∼4	2148±2317	2301±3079	0.456
	4∼5	1975±1623	1953±1258	0.844
	5∼6	1871±1148	2046±2086	0.215
	0∼12	18947±11864	19104±11501	0.845
Before dialysis initiation (month)	−1∼0	3529±2696	4896±4629	<0.001
	−2∼−1	555±1297	1092±2624	<0.001
	−3∼−2	367±922	635±1779	0.015
	−4∼−3	333±1003	725±3092	0.032
	−5∼−4	259±705	382±1180	0.108
	−6∼−5	247±520	417±1352	0.042
	−6∼0	4926±3811	7162±6762	<0.001
	−12∼0	6206±5873	8610±7820	<0.001

Costs are expressed as the mean ± standard deviation values.

We evaluated the impact of various clinical and laboratory parameters on medical costs by univariate and multivariate analyses. Table 3 summarizes the results of the costs during the first month after dialysis initiation. The referral type (ER) reduced the costs (409.1±174.3 USD, P = 0.019) and the dialysis modality (hemodialysis) increased the medical costs (823.8±184.3 USD, P<0.001) during the first month in the univariate analysis. The patients' age, gender, body mass index, causes of renal disease, hemoglobin level, and serum albumin level were not associated with the medical costs. In the multivariate analysis, ER significantly reduced the medical costs (428.5±172.3 USD, P = 0.013) during the first month after dialysis initiation.

**Table 3:** Multivariate analysis for the medical costs during the first month after dialysis initiation.

	Univariate model		Multivariate model	
Variables	Parameter estimates	P Value	Parameter estimates	P Value
Type of referral (early referral)	−409.1±174.3	0.019	−428.5±172.3	0.013
Age (years)	−1.2±6.1	0.846		
Gender (female)	53.9±175.3	0.759		
Type of dialysis (hemodialysis))	823.8±184.3	<0.001	845.7±184.8	<0.001
BMI	−26.7±22.0	0.226		
Causes of renal disease				
Diabetes mellitus	−197.2±236.0	0.404		
Glomerulonephritis	−571.9±295.5	0.053		
Hypertension	−285.0±302.6	0.347		
Hemoglobin (g/dL)	−14.0±22.4	0.532		
Serum albumin (g/dL)	−4.1±6.1	0.503		

Parameter estimates (unit: USD) are expressed with β ± standard error values.

The univariate and multivariate analyses on the costs during the 1 year before dialysis also showed a significant association of the referral time with the medical costs (Table 4). In the univariate analysis, ER (2404.1±447.4 USD, P<0.001) and the cause of renal disease reduced the costs, and age (30.1±15.7 USD, P = 0.055) and hemoglobin level (89.9±47.3 USD, P = 0.057) showed an association with increased medical costs. In the multivariate analysis, ER was also proved to be significantly associated with reduced medical costs (2534.0±436.2 USD, P<0.001).

**Table 4:** Multivariate analysis for medical costs during the 1 year before dialysis initiation.

	Univariate model		Multivariate model	
Variables	Parameter estimates	P Value	Parameter estimates	P Value
Type of referral (early referral)	−2404.1±447.4	<0.001	−2534.0±436.2	<0.001
Age (years)	30.1±15.7	0.055	27.9±15.7	0.076
Gender (female)	−431.4±449.8	0.338		
Type of dialysis (hemodialysis)	389.2±478.5	0.416		
BMI	8.1±55.3	0.883		
Causes of renal disease				
Diabetes mellitus	−637.6±571.5	0.265	−860.2±596.7	0.150
Glomerulonephritis	−2520.9±720.0	<0.001	−2247.6±749.5	0.003
Hypertension	−1507.3±717.3	0.036	−1814.4±737.1	0.014
Hemoglobin (g/dL)	89.9±47.3	0.057	81.4±46.3	0.080
Serum albumin (g/dL)	7.2±8.8	0.412		

Parameter estimates (unit: USD) are expressed with β ± standard error values.

### Medications and Vascular Access

Frequency of medication use, including antihypertensive drug, antiplatelet agents, anticoagulants, vitamin D, and phosphate binders, were not different between the two groups. Erythropoiesis-stimulating agents use was more (68.0 vs. 59.3%, P = 0.007) in the ER group. In the ER group, frequency of native arteriovenous fistula was higher (30.7% vs. 20.1%, P = 0.003) and temporary tunneled-catheter use was lower (40.2% vs. 49.0%, P = 0.031).

## Discussion

We evaluated the benefit of ER to a nephrologist with respect to economic costs in a prospective cohort of patients who started dialysis for ESRD. We found that ER to a nephrologist significantly reduced medical costs during the periods of 1 year before and 1 month after the initiation of renal replacement therapy using national health insurance claim data. Patients with advanced CKD are frequently complicated with anemia, electrolyte imbalances, hypertension, mineral bone disorders, and other risk factors of cardiovascular diseases. Therefore, correcting and reversing the causes of renal impairment and managing the comorbidities in CKD are important to improve clinical outcomes and patient survival. Education of CKD patients about dialysis can reduce the incidence of emergent hemodialysis via a central venous catheter [38], [39]. Recently, we reported that the timely referral of patients with CKD to a nephrologist is associated with the reduced usage of a central venous catheter for hemodialysis, improved patient survival, and decreased cardiovascular mortality [32]. The participants of this study are slightly different from our previous investigation in that the duration of patients' enrollment of this study was shorter by about 4 months and the definition of the ER group was defined as patients who had been referred 1 year before dialysis and who had visited nephrology clinic at least two times irrespective to the status of education about dialysis. In this study, we also proved that early referral to a nephrologist could reduce the usage of temporary vascular catheter for hemodialysis, and improve patients' blood pressure and lipid level control. The economic benefits of ER to a nephrologist were expected due to reduced morbidities and improved patient survival. To date, there have been several reports that ER to a nephrologist is cost-effective. However, those studies were retrospective analyses or included indirect estimations of medical costs [21], [25], [40], [41]. In this study, we directly proved the cost-saving effect of ER by the integration of the clinical data of patients who started dialysis for ESRD from a prospective cohort and the claim data of a national health insurance organization.

Despite the various benefits of early nephrology referral, there are still many patients who are referred to a nephrology clinic too late for impending renal replacement therapy. The problems related to LR are common in many countries, and the proportion of patients who are referred when facing the impending dialysis is up to 31–38% [9], [42], [43]. The LR of CKD patients to a nephrologist can increase the medical costs due to increased mortality and comorbidities. The increased incidence of emergent hemodialysis and the frequent use of a temporary central venous catheter for hemodialysis contribute to the increased medical costs associated with LR [23], [44]. Moreover, the increased duration of admission for the first dialysis is an important factor for the increased economic burden [16]. Although we could not compare the duration of admission between the two groups, more patients in the ER group started hemodialysis with their own native arteriovenous fistula without insertion of temporary central venous catheter. Reduced catheter usage contributed to the cost-saving effects in the ER group. The increased medical costs in the LR group may have resulted from the increased number or severity of the comorbidities associated with rapidly aggravating renal dysfunction. However, in this study, the cost-saving effects of ER were independent of comorbid conditions because we could not find a difference in the severity of illness between the ER and LR groups. Cost-saving effects of ER did not persist more than 1 months after dialysis start. It can be possible that main factors influencing on the decreased medical costs of ER work on during the predialysis period or early period of dialysis initiation. Detrimental effects of LR have been reported to be influential on 3 months short-term mortality, but not on long-term mortality [45]. After dialysis start, all patients are treated by nephrologists, patterns of clinical practice affecting on medical costs necessarily come to be similar between the ER and LR groups. Interestingly, medical costs in the ER groups were remarkably decreased before dialysis start. Advanced CKD patients are complicated with various medical problems, including hypertension, dyslipidemia, diabetes, and mineral bone disease. If patients were not referred to a nephrologist at the right time, inappropriate or overlapped laboratory tests would increase the medical costs in the LR group. In addition, detailed and appropriate medical therapy in the ER group would decrease the morbidities and medical costs before dialysis initiation. In this study, although more patients in the ER group were prescribed erythropoiesis-stimulating agents and the usage of antihypertensive drugs was not different between the two groups, blood pressure and lipid level were better controlled and medical costs were decreased through cost-effective care in the ER group.

There are several causes of the LR of patients with CKD to a nephrologist despite the clinical benefits of ER. With respect to clinicians or medical facilities, the lack of communication between doctors or hospitals, the absence of definite guidelines for the referral time to a nephrologist, and ambivalent opinions about dialysis in the elderly or high risk patients are factors that affecting LR [46]. With respect to patients, the refusal of dialysis, a lack of knowledge about CKD and dialysis, and economic poverty might function as factors that affect LR. Economic difficulties have been reported several times as the main cause of LR [29], [47]. When patients with CKD are referred late due to economic difficulties, adequate treatment and preparation for dialysis are inevitably delayed. Increased metabolic complications related to ESRD eventually raise the medical costs related to dialysis and hospital admission, which in turn deteriorate the patients' economic status. The timely referral of patients with CKD, especially those with a poor socioeconomic status, to a nephrologist is necessary to prevent delayed dialysis due to a lack of knowledge and negative thinking about renal replacement therapy and to reduce the unnecessary medical expenses related to delayed referral.

Despite the unique findings and advantages, this study has several limitations. At first, there can be concerns about the lead time bias that patients in the ER group had better renal function, lower blood pressure, less severe anemia, and lower LDL cholesterol levels at the time of referral, and these reduced comorbidities could influence to the reduced medical costs in the ER group. These facts that patients in the ER group showed better clinical conditions are not surprising because they were referred about 5 years earlier than those in the LR group. At least, almost all parameters including age and co-morbidity index at the time of dialysis were similar between the ER and LR groups, and it is hard to say that patients in the ER group had better renal function or lesser comorbidities at the same time 6 or 12 months before dialysis initiation. If we could adjust parameters at the 12 months before dialysis instead of parameters at the time of dialysis initiation, multivariate regression model would be more accurately fitted to predict medical costs during the 12 months before dialysis initiation. However, due to the limited data collection in this cohort study, we could not gather clinical parameters or laboratory data at the 12 months before dialysis initiation, but only get data at the time of referral and at the time of dialysis initiation. Secondly, we only analyzed and compared the medical costs during the 12 months before and after dialysis initiation, not the whole or average medical costs from referral to dialysis initiation. Considering the 5 year mean duration from referral to dialysis in the ER group, if we could have compared the total 5 year medical costs or yearly medical costs during the 5 years between the two groups, the cost-saving effects of ER would be proved apparently without bias. The reasons why we focused on the medical costs during the 12 months before dialysis initiation were that we had divided the ER and LR groups by the referral time of 12 months and aimed to compare the medical costs around the time just before and after dialysis initiation. Actually, during the 12 months before dialysis initiation, monthly medical costs and the difference of medical costs between the ER and LR groups increased steadily. A considerable part of cost-saving effects of ER derived from the reduced medical costs during the several months of impending dialysis. It is still important that patients in the LR group spend much more medical costs probably due to the increased length of hospital stay and increased use of temporary vascular catheters immediately before dialysis initiation. Lastly, we could not investigate the relationship between medical costs and other clinical and laboratory parameters including eGFR, co-morbidity index, blood pressure, and cholesterol levels in univariate analysis. However, it might not lessen the strength of our results because these parameters were similar between the two groups.

The care of patients with CKD requires not only evaluation and treatment of decreased renal function but also a multidisciplinary approach, including proper blood pressure control, appropriate selection of medications and their dosages, the avoidance of nephrotoxic drugs, education about diet, preparation for renal placement therapy, psychological consultations, and economic evaluations. Timely referral is essential to improve patient outcomes and survival, but a considerable proportion of patients are referred late to dialysis for various reasons, including a lack of knowledge, negative preconceptions about dialysis, and economic difficulties. In this study, we demonstrated that the ER of CKD patients could reduce economic costs during the period before and after the initiation of renal replacement therapy. Considering the benefits of ER to a nephrologist in both economic and clinical terms, clinicians should focus on the timely referral of patients with CKD.
